# Infrared Spectroscopic Profiling of Volatile Metabolites from Uropathogenic Bacteria: Basic Investigations toward Rapid UTI Diagnostics

**DOI:** 10.1021/acs.analchem.5c03269

**Published:** 2025-09-25

**Authors:** Kiran Sankar Maiti, Susmita Roy, Christian Zenner, Lindsay J Hall, Jürgen Hauer, Ronald Sroka

**Affiliations:** † TUM School of Natural Sciences, Department of Chemistry, 9184 Technical University of Munich, 85748 Garching, Germany; ‡ Laser-Forschungslabor, LIFE-Center, 9183 LMU University Hospital, LMU Munich, 82152 Planegg, Germany; § TUM University Hospital Rechts der Isar, 155892 Technical University of Munich, School of Medicine and Health, Ismaninger Str. 22, 81675 Munich, Germany; ∥ LMU Munich, Department of Veterinary Sciences, Chair of Bacteriology and Mycology, Sonnenstraße 24, 85764 Oberschleißheim, Germany; ⊥ School of Life Sciences, Intestinal Microbiome, 9184 Technical University of Munich, Weihenstephaner Berg 3, 85354 Freising, Germany; # Department of Microbes, Infection and Microbiomes, School of Infection, Inflammation and Immunology, College of Medicine and Heath, University of Birmingham, Birmingham B15 2TT, U.K.; ∇ Department of Urology, LMU University Hospital, LMU Munich, 81377 Munich, Germany

## Abstract

Urinary tract infections (UTIs) are among the most common bacterial infections, typically begin in the urethra but capable of rapidly progressing to the bladder and kidneys. Without timely intervention, these infections can lead to renal failure and, in severe cases, multiorgan failure. Current diagnostic methods for identifying bacterial pathogens are often time-consuming, highlighting the need for faster, more efficient detection techniques. Metabolite-based bacterial diagnostics offer a promising alternative, enabling minimally or noninvasive detection without the need for bacterial culture—provided that the metabolic profiles of individual bacterial strains are well characterized. This study investigates ten bacterial species associated with UTIs through the analysis of their volatile metabolites using Fourier-transform infrared (FT-IR) spectroscopy. Numerous spectral features corresponding to distinct metabolites were identified. Within a multidimensional metabolic space, each bacterial strain exhibited a unique volatile metabolite profile, serving as the basis for accurate identification. This approach lays the groundwork for future diagnostic platforms in which FT-IR spectroscopy could serve as a rapid, culture-free analytical tool to detect bacterial pathogens directly from exhaled breath and/or urine.

## Graphical abstract

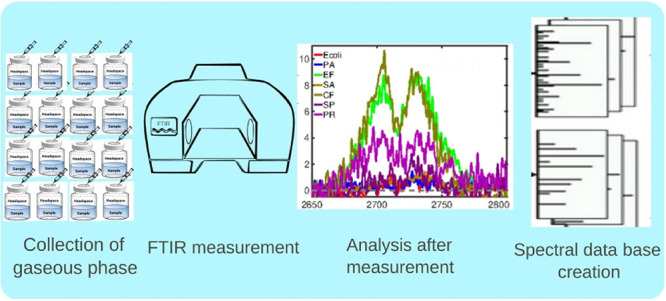

## Introduction

Urinary tract infections (UTIs) are among the most common bacterial infections and pose an increasingly serious public health threat.^1,2^ Despite significant advancements in medical care to combat bacterial infections, the number of UTI cases has risen by nearly 70% over the past 30 years.^3^ In 2019, an estimated 400 million people worldwide were affected by UTIs, resulting in approximately 240,000 deaths. UTIs have a profound impact on well-being, quality of life, economic burden, and cause psychological stress.^3,4^ Incidence statistics indicate that women are most affected due to their anatomical structure; however, especially older adult men also frequently experience UTIs.^5–7^ A UTI typically begins with periurethral contamination, but pathogens can rapidly migrate to the bladder and subsequently to the kidneys. Without timely intervention, this can lead to renal failure and sepsis.

To enable prompt intervention, patients with symptomatic UTIs are often treated with antibiotics before the causative bacteria are identified.^2^ However, this approach may not fully prevent recurrence and can lead to long-term alterations in the normal microbiota, as well as the emergence of multidrug-resistant microorganisms.^8,9^ This, in turn, increases the risk of colonization by multidrug-resistant uropathogens, potentially resulting in immunosuppression.^10,11^ Therefore, rapid and accurate identification of the causative bacteria is crucial for effective UTI treatment.

The current state-of-the-art method for identifying UTI-causing bacteria primarily relies on urine cultures.^12^ However, this culture-based approach is labor-intensive, time-consuming (requiring at least 72 h), and subject to several preanalytical limitations that can compromise diagnostic accuracy. Genome sequencing is increasingly adopted in public health settings to enhance diagnostic accuracy; however, it still depends on pure cultures, sequencing, and analysis, offering little to no time advantage over traditional methods.^13^ Furthermore, contamination during urine culture collection is a common issue, leading to inappropriate antibiotic use, misdiagnosis, and unnecessary exposure to toxic treatments. Imaging techniques based on Raman spectroscopy provide rapid differentiation between noninfectious systemic inflammatory response syndrome and sepsis; however, they are unable to identify bacterial and fungal infections.^14,15^ Given these challenges, developing culture-free, rapid pathogen detection techniques are essential for improving UTI diagnosis and treatment.

Diagnosis based on metabolites, which are characteristic chemicals in living organisms, appears to be a promising approach for overcoming the challenges of UTI bacterial identification.^16–19^ Within every living system, numerous biochemical reactions occur to sustain life. These biochemical processes, collectively known as metabolism,^20–22^ are highly specific, generating distinct metabolites unique to their respective pathways.^23^ By analyzing bacterial metabolites, it seems possible to achieve definitive bacterial identification.^24–26^

When pathogens colonize the host, they draw nutrients from host tissues and release abundant metabolic byproducts into the circulatory system. Consequently, bacterial metabolites can be found in the host’s biofluids.^27–29^ Analyzing these biofluids facilitates bacterial identification and characterization of population dynamics, provided that bacterial metabolism and its interaction with host metabolism during infection are well understood.^30,31^ Biofluids such as blood, urine, and saliva are metabolite-rich and routinely analyzed for various diagnostic purposes. However, bacteria-specific metabolites, which are present in trace amounts, are challenging to detect in liquid phase. In contrast, small metabolites in the gas phase are comparatively easier to identify.^32–35^ In this regard, exhaled breath and urine headspace may serve as the most promising sources of bacterial metabolites.

Several analytical approaches have shown promise for the identification and quantification of volatile metabolites, commonly referred to as volatile organic compounds (VOCs). Techniques such as mass spectrometry,^36^ electronic nose (e-nose),^37^ infrared spectroscopy,^38,39^ and photoacoustic spectroscopy^40^ have demonstrated their potential for VOC analysis. However, each method has its own advantages and limitations regarding reliability, cost-effectiveness, system size, analysis time, and user-friendliness.^41,42^ For instance, mass spectrometry has made the most significant contributions to VOC analysis; however, due to issues with reproducibility, its large system size, and extremely high cost, it is not yet suitable as a clinical diagnostic tool.^43^ In contrast, e-nose technologies are very promising due to their compact size and affordability, but their inability to identify specific VOCs, as well as concerns about reliability, require extensive testing and further refinement.^44^ Infrared spectroscopy, on the other hand, emerges as a promising alternative, offering rapid, reliable, cost-effective, and user-friendly analysis of gaseous metabolites.^45,46^

Gaseous biomarker analysis using infrared spectroscopy is an emerging diagnostic approach for disease detection, and the infrared spectroscopic features of many biomarkers are already known.^47^ However, its application in bacterial identification is relatively new,^48,49^ and the infrared spectral profiles of bacterial metabolites are not yet well understood. A recent proof-of-principle study demonstrated that infrared spectroscopy can identify and differentiate pathogens by analyzing their volatile metabolic components.^26^ However, to identify UTI-associated bacteria through host breath or urine, the infrared spectral profiles of the relevant pathogens must first be established. The unique infrared spectral signature of each bacterial species enables subsequent identification of UTI-causing bacteria in exhaled breath or urine. This work provides spectral profiles of cultured bacterial species that are commonly responsible for UTIs, thereby providing a foundational reference for future translational studies.

## Experimental Methods

### Bacterial Selection and Culture

The following bacteria were selected from MIQ 02: Urinary Tract Infections: *Escherichia coli* WS1322 ( *E. coli* ), *Enterococcus faecalis* WS2439 ( *E. faecalis* ), *Pseudomonas aeruginosa* G10837 ( *P. aeruginosa* ), *Staphylococcus aureus* WS2286 (*S. aureus*), *Morganella morganii* (*M. morganii*), *Providencia rettgeri* (*P. rettgeri*), *Citrobacter freundii* ( *C. freundii* ), *Streptococcus pyogenes* ( *S. pyogenes* ), *Citrobacter koseri* (*C. koseri*), and *Enterobacter cloacae* (*E. cloacae*). Among these, *E. coli* is the most prevalent pathogen causing UTIs. The first four bacterial strains were obtained from the Weihenstephan in-house culture collection, while the remaining six were collected from the department of Medical Microbiology, LMU.

Bacteria were cultured on Trypticase Soy Yeast Extract Agar (30 g/L trypticase soy broth, 3 g/L yeast extract, and 15 g/L agar) at 37 °C for 24–48 h. To ensure purity of the isolates, single colonies were restreaked three times. A single colony was then transferred to 25 mL of Trypticase Soy Yeast Extract Broth (TSB) in Falcon tubes and incubated at 37 °C for 24–48 h. Subsequently, 1 mL of the incubated TSB was transferred into 300 mL of TSB in 500 mL Schott bottles equipped with a punch-through cap and a silicone septum, followed by incubation at 37 °C for 24–48 h. All bacterial cultures were prepared in triplicate. Negative controls consisted of 1 mL of TSB added to culture bottles without bacteria.

### Headspace Collection

The headspace (bacterial breath) from each bacterial culture was collected for gas-phase metabolic analysis. The sample collection procedure has been previously described.^26^ A 250 mL glass syringe was used for the headspace collection. Two 20G cannulas were inserted through the silicone septum—one with a long catheter and the other short. The long cannula was pushed in such a way that the needle remains just above the culture broth. The syringe was connected to the short cannula to collect the bacterial headspace, while the long cannula prevented underpressure by allowing air to enter during sampling. The headspace from each culture bottle was withdrawn four times to fill 1 L TEDLAR bags for gas sampling. All samples were collected simultaneously and immediately sent for spectroscopic measurement, which was performed on the same day. The list of urological bacteria used for headspace analysis and number of measurements performed for each bacterium are presented in Table 1.

**Table 1:** List of Urological Bacteria Used for Headspace Analysis and Number of Measurements Performed for Each Bacteria^a^

	number of replicates			Gr.
bacteria	day I	day II	total	
*Escherichia coli* (*E. coli*)	2	1	3	FAA
*Enterococcus faecalis* (*E. faecalis*)	2	1	3	FAA
*Pseudomonas aeruginosa (*P. aeruginosa*)*	2	1	3	A
*Staphylococcus aureus* (*S. aureus*)	2	2	4	FAA
*Enterobacter cloacae* (*E. cloacae*)	2		2	FAA
*Morganella morganii (*M. morganii*)*	2	1	3	FAA
*Providencia rettgeri* (*P. rettgeri*)	2	1	3	FAA
*Citrobacter freundii* (*C. freundii*)	2	1	3	A
*Streptococcus pyogenes (S.pyogenes)*	2	1	3	FAA
*Citrobacter koseri* (*C. koseri*)		3	3	FAA
*negative control*		3	3	

a Gr. stand for classification based on their oxygen requirements. A: Aerobic - require oxygen for survival; FAA: Facultative anaerobic - can survive with or without oxygen.

### Sample Preparation

In principle, the collected sample can be measured as it is. However, due to the high water vapor content in the headspace sample, the infrared spectral features of bacterial metabolites are obscured by water absorption spectra. In the presence of water vapor, obtaining meaningful metabolic information from bacterial breath is practically impossible. Therefore, removing water vapor as much as possible is essential to generate clear and interpretable absorption spectra. Recent advancements in water suppression techniques for gaseous samples^50^ have addressed this challenge. A detailed sample preparation method for bacterial breath has been previously described,^26^ with a brief overview provided here.

The water-suppressed samples were prepared by freezing water vapor at −60 °C in a home-built cold trap. A 12-m-long copper tube (ID = 3 mm, OD = 6 mm) was coiled into a spiral configuration and placed inside a sealed metal chamber filled with a silicon-based bath fluid, which operated at temperatures ranging from −95 to +55 °C. The chamber’s temperature was precisely controlled using an ultralow refrigerated circulator (FW95-SL, Julabo Labortechnik GmbH).

Before sample preparation, the entire system was evacuated to a pressure of 10^–5^ mbar while maintaining the bath fluid temperature at 45 °C. This high temperature ensured the removal of any residual contaminants from previous experiments within the sample flow path. After evacuation, the bath fluid temperature was lowered to −60 °C, and the collected bacterial headspace was introduced into the sample preparation unit.

With a controlled flow rate of 3 mL/sec, bacterial breath was directed through the cold coiled copper tube into the evacuated sample cell connected to the FTIR spectrometer. The controlled flow ensured that the water vapor remained within the cold spiral long enough to freeze, achieving a three-order reduction in water vapor content without compromising valuable metabolites. Finally, the water-suppressed samples were transferred to a sample cell with a 4-m optical path length and a 2-L volume (Bruker Optics GmbH, Germany) for measurement. To ensure uniformity, the sample cell was filled to 500 mbar.

### Spectroscopic Measurements

The infrared absorption spectra of bacterial breath were measured using a Fourier transform infrared (FTIR) spectrometer (Vertex 70, Bruker Optics GmbH, Germany). Spectra were acquired over a spectral range of 500–4000 cm^–1^ with a resolution of 0.5 cm^–1^, utilizing a liquid nitrogen-cooled mercury cadmium telluride (MCT) detector.

During measurements, both the spectrometer and sample chamber were purged with dry nitrogen to eliminate water vapor from the optical path. Despite maintaining a steady nitrogen flow, slight fluctuations in residual water molecules within the spectrometer were expected. To minimize variations in water vapor concentration between background and sample scans, a background spectrum was recorded immediately before each sample measurement. This approach significantly improved data quality.

To further reduce background noise, 100 spectra were collected and averaged for each sample, a process that typically took about 5 min. Under optimal conditions, the entire measurement, including sample preparation and system cleaning, required approximately 20 min. For consistency, a fixed volume of one liter of headspace sample was used for each measurement.

The estimated limit of detection (LOD) for a one-liter gaseous biofluid sample was 10 parts per billion (*ppb*) for metabolites such as carbon monoxide, methane, and acetone.^46^ Notably, the LOD of the measurement system is inversely proportional to the sample volume. For example, reducing the sample volume to one-fourth would increase the LOD by a factor of 4.

### Spectroscopic Data Analysis

All infrared spectra were analyzed using the MATLAB R2024a. In the absorption intensity profiles of bacterial metabolites, the spectral baseline exhibited not only a linear shift along the intensity axis but also angular deviations and nonlinear distortions. To address these issues, hierarchical baseline corrections were applied to each data set prior to spectral analysis. A detailed description of the baseline correction procedure is available elsewhere;^51^ here, a brief overview is provided. As a first step, the linear shift was corrected by aligning the molecular silent region (∼2500 to 2700 cm^–1^) of the infrared spectra with the zero-intensity baseline. Next, angular deviations were corrected by rotating the spectrum about the zero-intensity line, according to the following equation:

$${\mathrm{AS}}_{i}=y_{i}-\frac{y_{N}-y_{1}}{N}(N-i)\;500\leq i\leq 4000$$ 1

where AS_*i*_ represents the angular shift at the *i* ^th^ point, *N* is the total number of data points, and *y*_1_ and *y*_*N*_ are the first and last data values in the selected data set, respectively. Finally, nonlinear baseline distortions were corrected segment-wise, under the assumption that within sufficiently small spectral windows, the baseline can be approximated as linear.

Following baseline correction, the infrared spectra were examined to identify significant spectral features. These observed features in bacterial samples were then compared with gas-phase molecular spectra using least-squares fitting to achieve optimal agreement.^46^ Typically, gas-phase molecular spectra were sourced from commercial databases such as PNNL,^52^ HITRAN,^53^ and NIST44,^54^ or obtained experimentally and theoretically through quantum chemistry calculations.^46,55^

## Results and Discussions

All infrared spectra presented here are the average of three replicates for nine bacterial strains and four replicates for one strain. Prior to averaging, individual spectra for each strain were carefully examined to ensure the absence of experimental artifacts or bacterial contamination, and to confirm that the spectra were consistent across replicates. For example, the average spectral feature of acetone is shown in Figure 1, together with the spectral span of all *C. koseri* replicates. The shaded region represents the spectral span, which deviates significantly small amount from the average spectral feature of acetone. The calculated standard deviation of each measured data point is indicated by the blue line. The consistently low standard deviation (SD) and the mean value of that (σ) demonstrates the high reproducibility of the bacterial replicates. As an additional control for uncontaminated bacterial growth, all three replicates of the negative control were also inspected.

**Figure 1:**
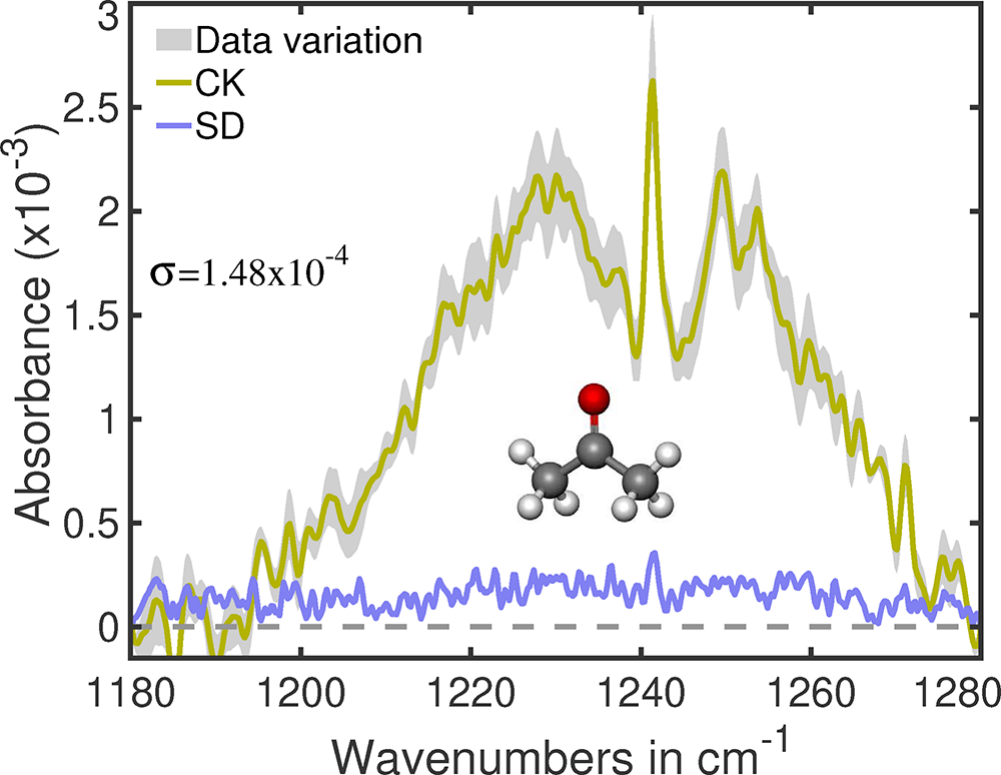
Average absorbance spectrum of acetone from the bacterial headspace of *C. koseri* (CK), with standard deviation (SD) and data variation shown. The colors of the spectral lines are indicated in the figure. σ indicates mean standard deviation.

The infrared absorption spectra of ten bacterial headspaces are shown in Figure 2a, covering a spectral range of 800–3450 cm^–1^. Each spectrum is color-coded uniquely for each bacterium, with this color scheme consistently maintained throughout the article. Spectra from the “negative control” is plotted with light gray line. The spectra exhibit numerous features, some shared among multiple bacteria and others unique to specific strains. Even though many bacteria share some spectral features, their absorption strengths vary, indicating differences in the production levels of specific metabolites. This variation could be due to two factors: (1) differences in bacterial population growth rates or (2) variations in metabolic rates for producing a particular metabolite. Although the spectra in this figure may appear congested and challenging to interpret, subsequent analysis demonstrates that multidimensional metabolite profiling is capable of accurately discriminating between bacterial strains.

**Figure 2:**
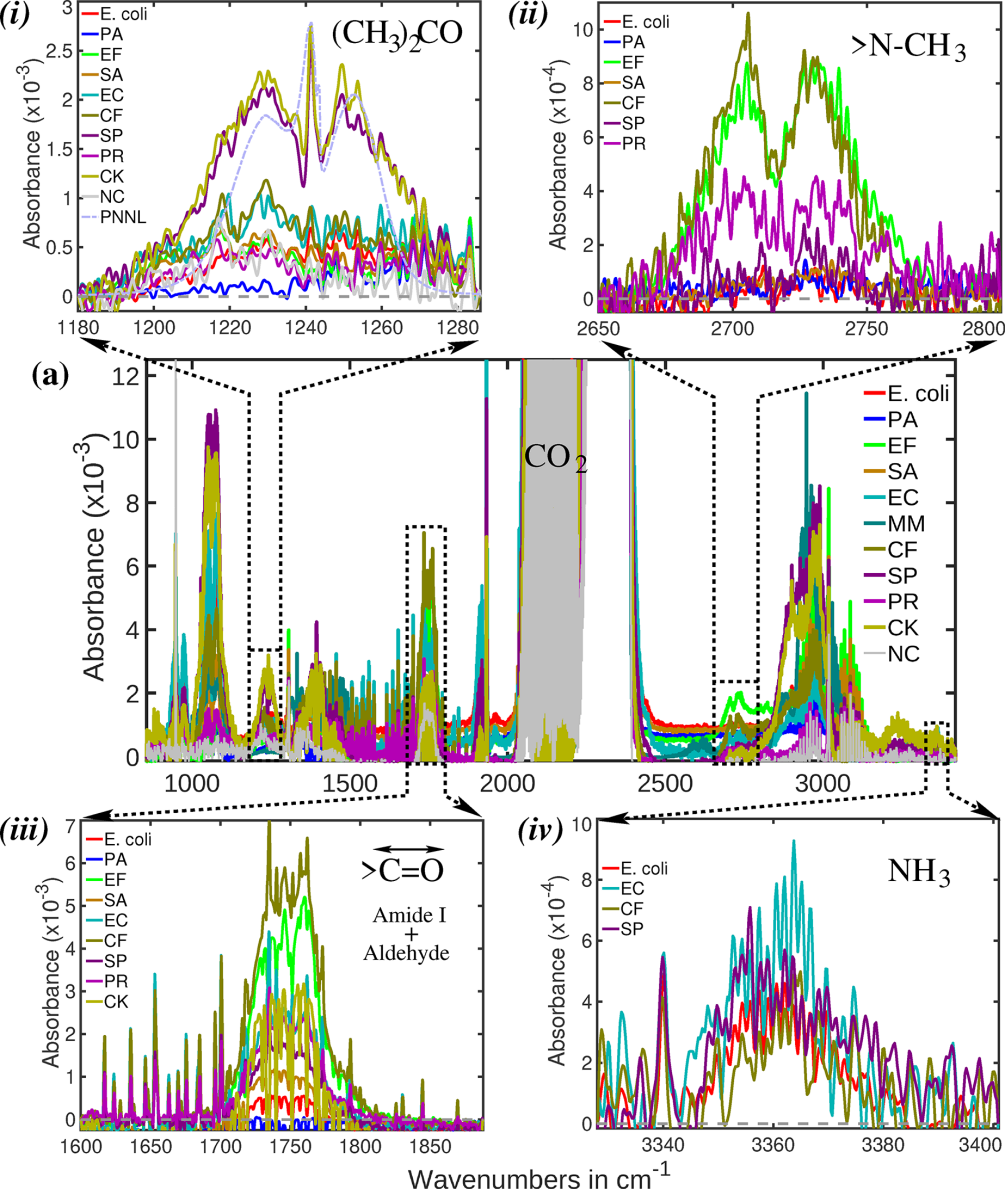
(a) Infrared absorption spectra of ten bacterial headspace causing UTIs: *E. coli* ( *E. coli* ), *E. faecalis* (EF), *M. morganii* (MM), *P. rettgeri* (PR), *C. freundii* (CF), *S. pyogenes* (SP), *C. koseri* (CK), *E. cloacae* (EC), *P. aeruginosa* (PA), and *S. aureus* (SA). NC denotes the negative control. Panels (i)–(iv) show magnified spectral regions to provide a clearer view of characteristic features of individual bacterial species. Additionally, panel (i) includes reference spectrum from the PNNL database (dotted light blue line), demonstrating a reasonably good match that confirms the presence of acetone in the bacterial headspace.

To improve spectral clarity, four regions have been magnified along both the wavenumber and absorption axes, as shown in Figure 2i–iv. These spectral features correspond to characteristic bacterial metabolites, namely acetone, aldehyde, ammonia, and trimethylamine. The following sections provide a detailed explanation of the spectral characteristics of these metabolites, along with additional metabolites not included in Figure 2.

### Carbon Dioxide (CO_2_)

A very strong absorption peak, which nearly saturates the infrared detector for many bacteria, is observed around 2350 cm^–1^. This absorption arises from the asymmetric stretching vibration of carbon dioxide^56^ (CO_2_). While CO_2_ is a common byproduct of bacterial metabolism, extracting meaningful information from this IR absorption peak is challenging, as it often leads to detector saturation. Instead, the bending mode and overtone absorption of CO_2_, which occur at much lower intensities, serve as more effective probes for bacterial identification using infrared spectroscopy. For instance, the CO_2_ absorption peak around 1050 cm^–1^ is particularly significant for distinguishing bacterial species by comparing the absorption strength for different bacterial species.^26^ A quantitative analysis of the CO_2_ peak is presented in Table 2 and discussed in section CO2 Combination Band.

**Table 2:** Area under the Curve for Each Spectral Features of Individual Bacteria Is Presented in Arbitrary Units (a.u.)^a^

	metabolites under investigation								
		CO_2_	alcohols	(CH_3_)_2_CO	amide	CO	TMA	CH_4_	NH_3_
bacteria	CW→	1050	1066	1240	1750	2175	2725	2950	3360
*E. coli*		182.3	0.0	34.1	22.3	429.1	1.2	243.6	9.6
*P. aeruginosa*		24.6	0.0	15.4	0.0	1105.2	6.3	113.5	4.2
*E. faecalis*		21.5	0.0	35.4	216.1	1190.0	49.0	272.4	5.3
*S. aureus*		105.6	0.0	37.6	41.6	1436.2	5.0	276.3	4.9
*E. cloacae*		248.2	0.0	56.8	136.2	660.7	36.5	243.9	16.2
*M. morganii*		51.8	0.0	11.0	9.5	92.8	3.3	74.9	6.8
*C. freundii*		137.7	0.0	50.4	295.0	1539.3	44.2	170.2	9.7
*S. pyogenes*		415.0	50.4	112.4	104.8	369.0	5.2	833.7	16.0
*P. rettgeri*		28.1	0.0	27.7	126.4	498.3	27.2	28.7	0.0
*C. koseri*		379.2	45.3	116.1	93.5	543.5	18.4	591.7	0.0
negative control		10.0	0.0	1.9	6.2	149.7	0.0	61.3	0.0

a For better realization, the original calculated values have been multiplied by 1000. ‘CW’ denotes the central wavelength of the spectral features, expressed in cm^–1^.

### Acetone ((CH_3_)_2_CO)

Figure 2i highlights a narrow spectral window between 1180–1280 cm^–1^. Within this region, spectral features vary notably among different bacterial species. A particularly distinct absorption feature is observed for the pathogens *S. pyogenes* and *C. koseri*. This feature corresponds to the C–C stretching vibration of acetone, characteristic of its molecular backbone.^57^ The reference spectrum from the PNNL database is plotted as a dotted light blue line, showing a reasonably good match that confirms the presence of acetone in the bacterial breath.

It is well established that certain anaerobic, acetogenic bacteria are capable of producing acetone through their metabolic processes, using CO_2_ and hydrogen (H_2_) as substrates.^58^ In the human gut, several acetogenic species have been identified that consume H_2_ produced during dietary fermentation.^59^ Although no studies have specifically addressed the presence of acetogenic bacteria in the urogenital microbiome, it is known that UTIs often result from the translocation of gut bacteria into the urogenital tract.^1^ This suggests that acetone could potentially serve as a biomarker for UTIs.

Among the bacterial species analyzed, *C. freundii* and *E. cloacae* also exhibit acetone-related spectral signatures, though their intensities are markedly lower than those of *S. pyogenes* and *C. koseri*. *E. coli* , the most common cause of UTIs, shows an even weaker acetone signal. In contrast, virtually no acetone signature is detected for *P. aeruginosa* . A quantitative assessment of acetone is provided in Table 2.

These findings suggest that acetone could serve as a potential biomarker for detecting specific bacterial pathogens associated with UTIs.

### Trimethylamine (TMA)

A second spectral region, centered at 2725 cm^–1^, is magnified and shown in Figure 2ii. In this region, distinct double absorption peaks are observed for *C. freundii* and *E. faecalis* . These features are likely attributed to the presence of trimethylamine—a compound produced by gut microbiota through the metabolism of dietary quaternary amines.^60–62^ Trimethylamine is a uremic toxin, with elevated levels detected in the urine of patients with UTIs.^63,64^ Additionally, trimethylamine has been associated with an increased risk of atherosclerosis, as well as serious cardiovascular diseases. However, due to its typically low abundance, its production by pathogenic bacteria and its broader implications in disease remain underexplored.^65^ Despite this, the prominent absorption features detected here highlight the potential of infrared spectroscopy for further investigation.

*P. rettgeri* also exhibits a spectral signature associated with trimethylamine, although its intensity is significantly lower than that observed in the other two pathogens (see Table 2). Interestingly, *C. freundii* displays a clear signature for both acetone and trimethylamine, whereas *E. faecalis* only shows the latter. Given these observations, trimethylamine may serve as an additional biomarker for identifying *C. freundii* . None of the other selected bacterial species exhibit a spectral signature indicative of trimethylamine.

### Amide-I (−CONH−)

Another spectral region of interest is the amide-I band, centered around 1750 cm^–1^ (see Figure 2iii), which primarily arises from the C=O stretching vibration of carbonyl groups.^66–68^ In gas-phase biofluids, this feature typically appears due to the presence of small peptides and/or aldehyde or ketone molecules. However, its signal is often significantly modulated by water absorption lines. Despite this, the varying absorption strengths observed across different bacterial headspaces suggest that distinct bacterial species produce differing amounts of peptides and/or aldehydes. A quantitative evaluation is presented in Table 2. For instance, *C. freundii* exhibits the strongest absorption peak in this region, whereas *P. aeruginosa* shows no indication of an amide-I band. *E. coli* , commonly associated with UTIs, shows only a weak absorption, implying it produces a relatively small amount of peptides or aldehydes through its metabolic processes. Other bacterial species display absorption peaks of varying intensities, enabling more accurate bacterial identification in multimetabolite analyses.

### Ammonia (NH_3_)

Ammonia is an important metabolite involved in various metabolic processes. However, not all bacteria rely on these pathways; thus, the presence or absence of ammonia can be used to differentiate between bacterial species. In infrared spectral analysis, ammonia exhibits a distinct absorption feature around 3360 cm^–1^ (see Figure 2iv), attributed to N–H stretching vibrations.^69–71^ Its H–N–H bending mode also generates a strong absorption near 1000 cm^–1^, however, this feature often overlaps with the CO_2_ absorption band, making it difficult to isolate. Among the selected bacterial species, *E. coli* , *E. cloacae*, *C. freundii* , and *S. pyogenes* show clear spectral signatures of ammonia. The quantitative measurements are summarized in Table 2. Typically, ammonia is produced through the metabolism of amino acids and other nitrogen-containing compounds.^72^ Excess ammonia production may directly irritate the urothelium, contributing to an inflammatory environment during UTIs. Several ammonia-producing bacteria have also been linked to UTIs.^73^ Therefore, ammonia has potential as a biomarker for bacterial identification.

### CO_2_ Combination Band

Almost all strains of human microbiota produce CO_2_, but the amount varies significantly depending on the metabolic pathways and population of each strain. As previously mentioned, the strongest CO_2_ absorption peak, resulting from the asymmetric carbon–oxygen stretch, often saturates the infrared detector, rendering it ineffective for bacterial detection. Instead, the combination band around 1050 cm^–1^ provides sufficient absorption strength to quantify individual bacterial strains.^53,56^

Figure 3 shows the CO_2_ combination bands from the headspace of different bacterial cultures. The well-defined spectral envelope, with peaks at 1050 and 1075 cm^–1^, clearly indicates the presence of CO_2_. The varying absorption strengths reflect the differing levels of CO_2_ produced by each strain. The headspace of the negative control, shown as a gray line, exhibits no CO_2_ absorption, confirming the absence of contamination in the bath fluid.

**Figure 3:**
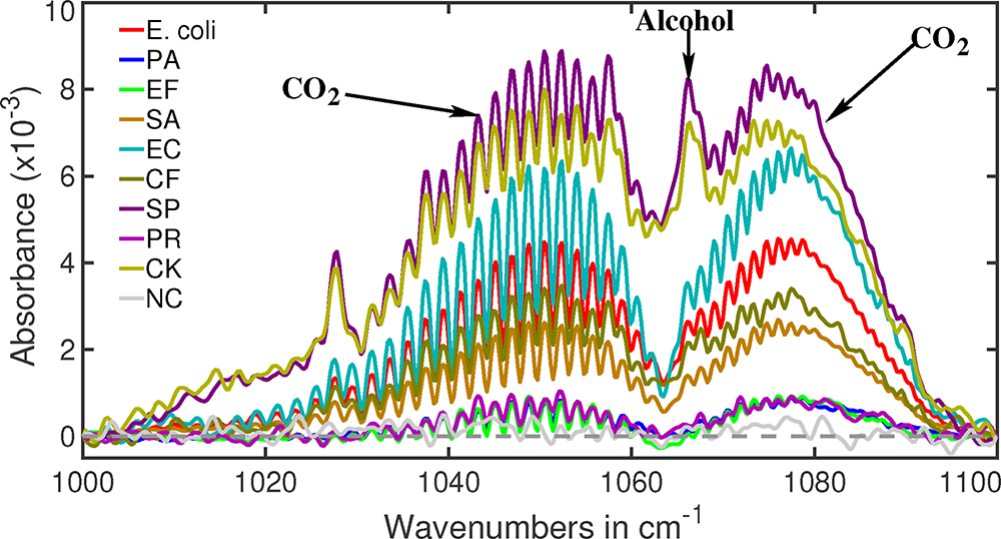
Absorption spectra of CO_2_ combination band of bacterial headspaces. Variations in absorption intensity indicate that different bacterial species produce differing amounts of CO_2_ as a byproduct of their metabolic activity. The small peak observed at 1066 cm^–1^ is a characteristic signature of alcohol. Among the bacteria studied, only *S. pyogenes* (SP) and *C. koseri* (CK) produce alcohol as a part of their metabolic processes.

Among the selected aerobic bacteria, *S. pyogenes* produces the highest amount of CO_2_, whereas *P. aeruginosa* produces the least. This suggests that either *S. pyogenes* has a higher growth rate or *P. aeruginosa* exhibits a lower metabolic activity to produce CO_2_. Other bacteria, regardless of their oxygen requirements, also produce varying amounts of CO_2_. This suggests that there is no direct relationship between a bacterium’s oxygen dependence and its CO_2_ production.

### Alcohol (−OH)

A particularly prominent spectral feature appears around 1066 cm^–1^ (see Figure 3), characteristic of the C–O stretching vibration typically observed in alcohols.^74,75^ Although alcohol production is uncommon among urological bacteria, recent studies suggest that some strains ferment ethanol as part of their metabolism.^76,77^ Among the bacteria studied, *S. pyogenes* and *C. koseri* show clear spectral signatures of alcohol fermentation, while the others do not (also see in Table 2). Thus, the presence of alcohol can serve as an important biomarker for bacterial identification.

### Carbon Monoxide (CO)

Carbon monoxide (CO) is a common byproduct for many biochemical processes.^23^ Some human pathogens produce CO as a byproduct of their metabolism as part of their energy-generating processes, and some can even use CO as an energy source.^78^ Therefore, in bacterial breath, amount of CO varies for different bacteria. In infrared spectroscopy, a distinct absorption feature is observed for CO centered at around 2175 cm^–1^. An oscillating absorption pattern between 2140 and 2220 cm^–1^ is well-known for CO identification.^50,53^ The infrared absorption spectrum of CO in bacterial headspaces are depicted in Figure 4. Practically absorption strength of CO varies 1 order of magnitude for selected bacteria. *M. morganii* shows lowest and *C. freundii* shows highest CO absorption strength.

**Figure 4:**
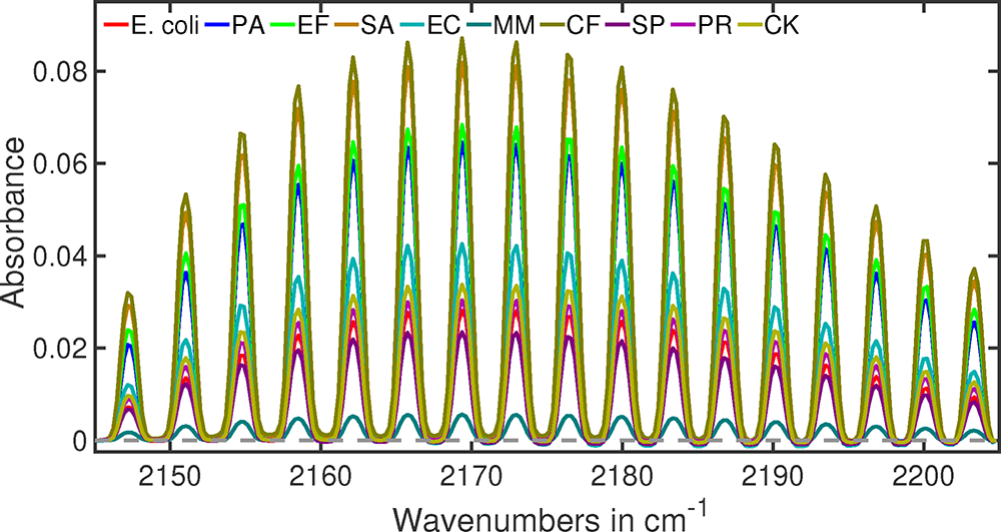
Infrared absorption spectra of carbon monoxide measured in bacterial headspaces.

### Methane (CH_4_)

Some bacteria produce methane as a byproduct of their metabolic activities.^79^ These methane-producing bacteria are commonly referred to as methanogens. In infrared spectroscopy, methane exhibits a characteristic spectral signature with well-defined **P**, **Q**, and **R** branches^50^ (see Figure 5a). The reference spectrum of methane, obtained from the PNNL database,^52^ is shown as light blue dotted lines. These absorption features primarily originate from C–H stretching vibrations.^80^ The absorption spectra of bacterial headspace samples are also presented in Figure 5a. While the **Q** and **R** branches of the reference methane spectrum align closely with those of the bacterial headspace spectra, a notable discrepancy is observed in the **P** branch region. Specifically, the methane **P** branch is superimposed on the spectral features of other metabolites.

**Figure 5:**
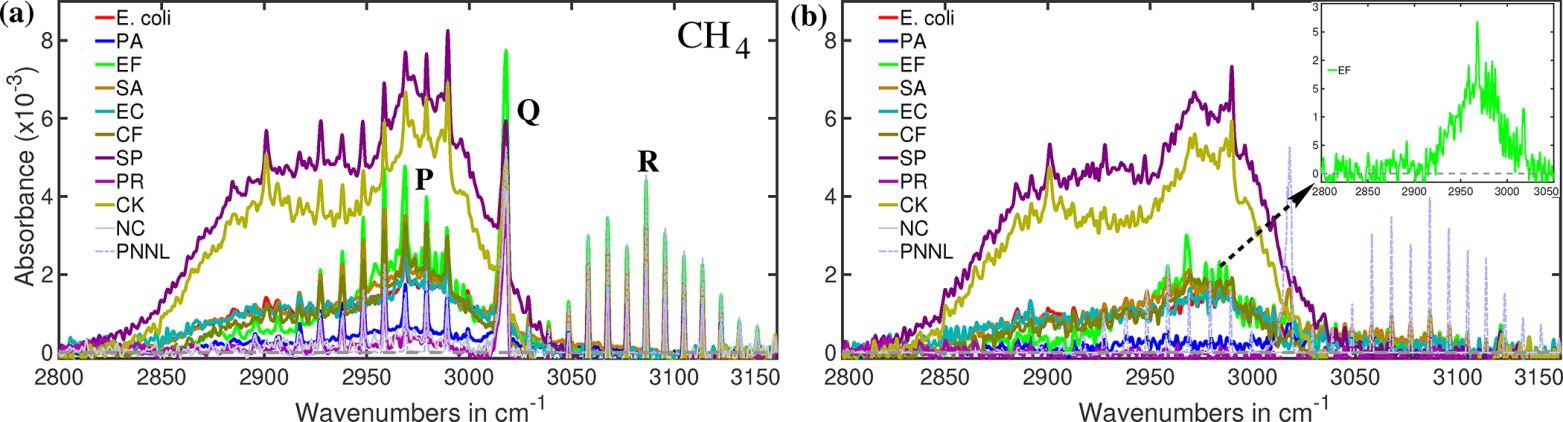
(a) Infrared spectra of the bacterial headspace in the C–H stretching vibrational region of methane. The **P** branch of methane shows significant modulation due to interference from other metabolic byproducts. (b) Absorption spectra of the headspace obtained after digital subtraction of the methane infrared signature. For clarity, the absorption spectrum of *E. faecalis* (EF) is shown in the inset.

To isolate the contribution of methane, digital spectral subtraction was performed, as illustrated in Figure 5b. Post-subtraction, the methane features are effectively removed, revealing distinct spectral signatures of unidentified metabolites. Among these, *S. pyogenes* and *C. koseri* exhibit the strongest and somewhat similar absorption features, with a slight variation near 2925 cm^–1^. This suggests the spectral profile may consist of multiple molecular components. A similar spectral pattern typically seen in esters. Similar spectral characteristics have previously been attributed to bacterial metabolites such as methyl butyrate.^17^

Other bacteria, including *E. coli* , *E. cloacae*, *C. freundii* , and *S. aureus*, display nearly identical spectral features, albeit with weaker absorption intensity. In contrast, *E. faecalis* produces a distinctly different spectral pattern, shown as an inset in Figure 5b, which does not correspond to ester-related features. While methane itself may not play a pivotal role in bacterial differentiation for selected bacteria, the variations in spectral patterns within the C–H stretching region and their respective intensities may offer a valuable basis for bacterial identification.

### Multi-Component Metabolite Profiling

An obvious question arises: when multiple bacteria exhibit the same spectral feature—such as the amide-I band—does that feature still contribute meaningfully to their identification? While it is true that examining the amide-I band alone may not be sufficient to distinguish bacterial strains, in a multidimensional metabolite analysis, each spectral band plays a crucial role in enhancing identification accuracy.

To support the above statement, a quantitative analysis was conducted on a selection of bacterial species. For each selected species, the area under the curve (AUC) was calculated for all relevant spectral features and presented in Table 2. For instance, both *C. freundii* and *E. faecalis* show strong absorption at the amide-I band, with *E. faecalis* displaying approximately 25% less intensity than *C. freundii* (see Figure 2). However, both species exhibit similar absorption strengths for TMA, making it easier to distinguish between them using just those two metabolites. Investigating another spectral feature—such as that associated with acetone—adds discriminatory power: *C. freundii* displays an acetone absorption intensity 50% more that of *E. faecalis* , improving identification confidence between these two species.

However, as the number of bacterial species increases, these two parameters become insufficient for accurate differentiation. To enhance discriminatory power, we introduce an additional spectral feature associated with acetone. *S. pyogenes* and *C. koseri* show nearly identical acetone absorption, both being approximately twice as strong as that of *C. freundii* . Interestingly, *S. pyogenes* shows only half the amide-I intensity of *C. freundii* , allowing for better selectivity among these four bacterial strain.

Adding the ammonia spectral feature further enhances bacterial differentiation. For instance, *E. faecalis* does not exhibit any detectable ammonia absorption, while *E. coli* and *E. cloacae* show distinct ammonia signals, despite being nearly undetectable in the trimethylamine spectral region.

Bacterial identification can be visualized by mapping the data into an multidimensional metabolite space. For simplicity, two representative projections in three-dimensional (3D) space are shown in Figure 6. Figure 6a displays the bacterial distribution in the Acetone–Methane–Ammonia space, while Figure 6b shows the CO_2_–Acetone–CO space. In Figure 6a, all bacterial species are clearly separated except for *S. aureus* and *E. faecalis* , which appear overlapping. However, in Figure 6b, these two species are distinctly separated. As more metabolite dimensions are considered, the separation between bacterial species becomes increasingly clear in the multidimensional metabolite space. It should be noted that normalization of sample volume is advantageous but not essential. In a multimetabolite space, the relative ratios of characteristic metabolites are the key factors for identifying individual bacteria. Thus, by incorporating multiple metabolite signatures across various spectral bands, it shows the potential that each bacterium can be identified with high confidence.

**Figure 6:**
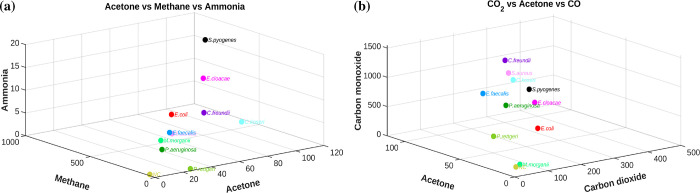
Position of bacteria in three-dimensional metabolite space. (a) Methane vs acetone vs ammonia and (b) acetone vs CO2 vs CO. All axes are scaled uniformly using arbitrary units.

It is well established that host biofluids are the primary source of pathogenic metabolites. Consequently, detecting pathogenic VOCs in exhaled breath and urine headspace is expected. When an infection develops in any part of the body due to pathogenic bacteria, it looks promising that their volatile metabolic signatures can be captured in the infrared spectra of these biofluids. However, accurate identification of the causative bacteria requires prior reference infrared spectral profiles. The present study represents an initial step toward developing such profiles for uropathogenic bacteria. Notably, the infrared analysis of gaseous biofluids with the current experimental procedure takes less than five minutes, promising that bacterial identification can become far more rapid than current diagnostic methods.

## Conclusions

This article presents a first step toward a rapid identification method for pathogens responsible for UTIs. The technique leverages the unique metabolic profiles of specific bacteria to identify individual strains. Infrared spectroscopy is employed to analyze volatile metabolites produced by the pathogens. Ten bacterial strains known to cause UTIs were individually cultured under well-controlled conditions, and their headspace gases were collected for infrared spectral analysis. Owing to the diversity of metabolic pathways among bacterial strains, each produces a distinct set of metabolic byproducts, which leads to characteristic features in the infrared absorption spectra. Several spectral features have been identified, analyzed and discussed as the basis for bacterial identification. While some features are shared among multiple strains, the overall metabolic fingerprint from multimetabolite analysis allows the definitive identification of bacterium by metabolite profiling.

Given the speed of infrared spectroscopic analysis compared to many other techniques, it holds significant promise for the development of rapid diagnostic tools that do not require bacterial culturing—provided that individual bacterial strains are well understood. Current efforts focus on understanding bacterial growth dynamics and building a comprehensive infrared spectral database of bacterial headspace profiles.

Despite its great potential as a patient-friendly and rapid diagnostic method, further improvements are required before it can be applied clinically. A laser spectroscopy–based multisensor diagnostic approach could significantly enhance detection sensitivity. However, the high dimensionality of human metabolites strongly influences the detectability of bacterial metabolites. Therefore, a thorough characterization of the metabolic interplay between bacteria and the host is essential.
